# Staining of palatal torus secondary to long term minocycline therapy

**DOI:** 10.4103/0972-124X.51896

**Published:** 2009

**Authors:** Aravind Buddula

**Affiliations:** *Department of Periodontics, Dental Specialties, Mayo Clinic, Rochester Minnesota - 559 05, USA*

**Keywords:** Minocycline, palatal torus, staining

## Abstract

Minocycline and other tetracycline analogs are well known to cause discoloring of alveolar bone, teeth and other tissues. The present case reports palatine torus discoloring, in a 91-year-old patient, after long term minocycline therapy. The patient was presented with staining of the palatal torus resulting from prior minocycline use for three-and-a-half years. The diagnosis of minocycline staining of palatal torus was done during a routine hygiene examination. The patient was informed that the bluish appearance of the palatal torus was the result of long term minocycline use. The patient was not willing to discontinue the antibiotic and was not concerned about the appearance. The clinician should inform patients on long term minocycline therapy about the possible side effects of staining of the alveolar bone, teeth and other soft tissue.

## INTRODUCTION

Several medications have been implicated in causing intraoral pigmentation, one of which is minocycline. It is a broad spectrum bacteriostatic antibiotic which inhibits protein synthesis. It is a drug commonly used to treat Acne vulgaris[1,2] and rosacea.[3] As with any medication, minocycline has side effects. Cutaneous pigmentation and discoloration of bones, teeth, thyroid tissue, nail beds, sclera and heart valves have been documented in literature.[4–6] This is a case report of a minocycline induced pigmentation of the palatal torus. Though many cases about the effects of minocycline on staining the alveolar bone have been documented, this case reports discoloration of the palatine torus in a 91-year-old patient following long term minocycline therapy.

## CASE REPORT

**Figure 1:**
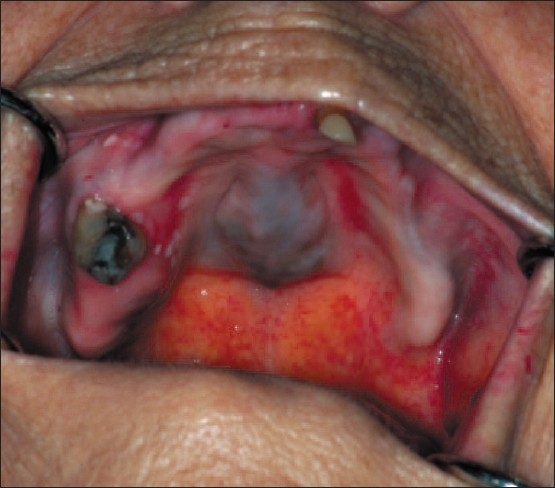
Staining of the palatal torus after minocycline therapy

A 91-year-old white female was presented to the Mayo Clinic for a routine hygiene appointment in July of 2007. The hygiene specialist was concerned about a blue discoloration on the hard palate. An intraoral examination revealed a palatal torus measuring 2.5 × 2cm with a blue discoloration [Figure 1]. There were no discolored areas noted intraorally.

A review of the medical history revealed that the patient had a three part intertrochanteric femur fracture in November of 2003. Subsequently, she underwent irrigation and debridement of her left hip due to a recurrent infection. It was then decided to indefinitely place the patient on minocycline 100 mg twice a day. The patient had been taking minocycline since then. The patient was seen on a regular maintenance schedule every six months. There was no discoloring on the palatal torus before July, 2007.

The discoloration of the palatal torus occurred approximately three-and-a-half years after the minocycline therapy was started. Based on the clinical appearance, given the long term use of minocycline, a diagnosis of minocycline-induced staining of the palatal torus was done. The patient was told that the condition may be reversible and the bluish discoloration might resolve if the minocycline is discontinued. The patient was not interested in discontinuing the antibiotics.

## DISCUSSION

A majority of the literature reported effects of minocycline staining on bone is in the form of case reports.[7–10] Westbury and Najera reported the incidence of minocycline staining of alveolar bone to be about 2% of the population taking the drug for two months or longer.[9] A literature search has also shown that the onset of discoloration can occur anytime from one month to many years after the initiation of treatment.[10,11] In this patient, minocycline staining of the palatal torus occurred approximately three-and-a-half years after minocycline therapy was instituted. Though clinically underlying bone appears blue or black, studies have shown that upon surgical exposure this bone has been described as being dark green,[12] dark gray-green,[13] greenish yellow[8] or gray in color.[14]

There is scant literature available about the minocycline staining the palatine torus.[11]

Most reports involve staining of the alveolar bone.

The minocycline staining may be the result of a slowly enlarging palatine torus and incorporation of minocycline into the newly formed bone. It is important for the clinician to realize that this condition is not a soft tissue condition but a manifestation of black pigmented alveolar bone showing through thin soft tissue. Even though the mucosa appears darkened, it is normal and should not be confused with other pigmented lesions of the oral cavity. Only one case report documented minocycline-induced discoloration of the oral soft tissues.[15]

Intraorally, minocycline use has also been associated with darkening of the crowns of fully erupted teeth, dark green discoloration of the roots of erupted teeth and black staining of the roots of developing teeth.[9] Unlike the staining of alveolar bone[16] or cutaneous pigmentation,[17] the discoloration of the permanent teeth does not necessarily resolve after discontinuation of minocycline therapy.[12,18]

A thorough review of the patient's medical history including the patient's medications is very important in cases where there is any intraoral staining of the alveolar bone or teeth. Patients should be informed that long term minocycline therapy could lead to staining of the teeth, alveolar bone and soft tissues. Patients should also be informed that, most times, staining is reversible with the discontinuation of antibiotic therapy.
